# Confirmation of cholera by rapid diagnostic test amongst patients admitted to the cholera treatment centre in Uvira, Democratic Republic of the Congo

**DOI:** 10.1371/journal.pone.0201306

**Published:** 2018-08-01

**Authors:** Aurelie Jeandron, Oliver Cumming, Baron Bashige Rumedeka, Jaime Mufitini Saidi, Simon Cousens

**Affiliations:** 1 Environmental Health Group, Department of Disease Control, Faculty of Infectious and Tropical Diseases, London School of Hygiene and Tropical Medicine, London, United Kingdom; 2 Ministère de la Santé Publique, Division Provinciale de la Santé Publique, District Sanitaire d’Uvira, Uvira, Sud-Kivu, République Démocratique du Congo; 3 Department of Infectious Disease Epidemiology, Faculty of Epidemiology and Population Health, London School of Hygiene and Tropical Medicine, London, United Kingdom; Johns Hopkins Bloomberg School of Public Health, UNITED STATES

## Abstract

**Introduction:**

Cholera is endemic in the Eastern provinces of the Democratic Republic of the Congo since 1978, and Uvira in South-Kivu has been reporting suspected cholera cases nearly every week for over a decade. The clinical case definition for suspected cholera is relatively non-specific, and cases are rarely confirmed by laboratory methods, especially in endemic settings. This may lead to over-estimation of cholera cases and limit effective public health responses.

**Methods and results:**

Between April 2016 and November 2017, 69% of the 2,059 patients admitted to the Uvira Cholera Treatment Centre (CTC) were tested for cholera with rapid diagnostic tests (RDTs). Of those admitted as suspected cholera cases, only 40% tested positive for cholera, equivalent to an estimated annual incidence of suspected/confirmed cholera in Uvira of 43.8 and 16.3 cases per 10,000 inhabitants respectively. A multivariable logistic regression indicates that boys aged 2 to 4 years, girls aged 5 to 15 years and adult men are respectively 1.9, 2.1 and 1.8 times more likely to test positive than adult women. On the contrary, boys under 2 are 10 times less likely to test positive. The odds of testing positive also increase as weekly admissions to the CTC rise, with up to a 5-fold increase observed during the weeks with the highest numbers of admissions compared to the lowest ones. Other predictors of cholera confirmation include duration of stay at the CTC, clinical outcome of admission, lower weekly rainfall and area of residence in Uvira, with the northern part of town having the highest confirmation rate.

**Conclusion:**

Cholera is an on-going public health problem in Uvira but the majority of suspected cases admitted to the CTC were found to be negative for cholera after RDT testing. These findings may have important implications for cholera control strategies in favour of interventions that address cholera and other diarrhoeal diseases alike.

## Introduction

In 2016, more than 130,000 cases of cholera were reported to the World Health Organisation (WHO) globally [1]. These figures are likely incomplete due to weak national surveillance systems and the actual global number of cholera cases is estimated to be closer to 3 million every year [2]. Comprehensive surveillance is also hampered by the relatively non-specific WHO standard case definition of suspected cholera cases, especially in endemic areas [3]. Enhanced surveillance data on the burden and characteristics of confirmed cholera across 11 study sites in seven countries of Sub-Saharan Africa by the AFRICHOL consortium show large variations in incidence and clinical presentation, with the proportion of confirmed cases amongst suspected cases ranging from 10% to 60% and a specificity of the WHO standard case definition of 8% [4, 5]. In Haiti, the specificity of this case definition in an outbreak setting was estimated to be only 43% over a two-year study in 4 hospitals [6]. Over-reporting of suspected cholera cases is therefore possible in areas with a long history of cholera outbreaks, when laboratory confirmation is only done on initial outbreak cases.

On the African continent, reported cholera incidence is generally higher inland than in coastal areas and, in the past two decades, outbreaks have been mostly clustered around the Great Lakes and Lake Chad regions [7]. Globally, increased cholera incidence and outbreaks tend to coincide with increased rainfall, although other climatic factors also appear to play a role, with seasonal patterns more pronounced further from the Equator [8].

With 28,093 suspected cases reported, the Democratic Republic of the Congo (DRC) was, in absolute numbers, the second most affected country after Haiti in 2016 [1, 9]. Provinces in the east of the country, particularly South-Kivu province, have been identified as a stable cholera transmission focus since 1978 [9, 10].

Our study describes the epidemiology of suspected and confirmed cholera based on admissions and results of rapid diagnostic testing collected over 18 months at the Cholera Treatment Centre (CTC) of Uvira in eastern DRC.

## Methods

### Study area

**Fig 1:**
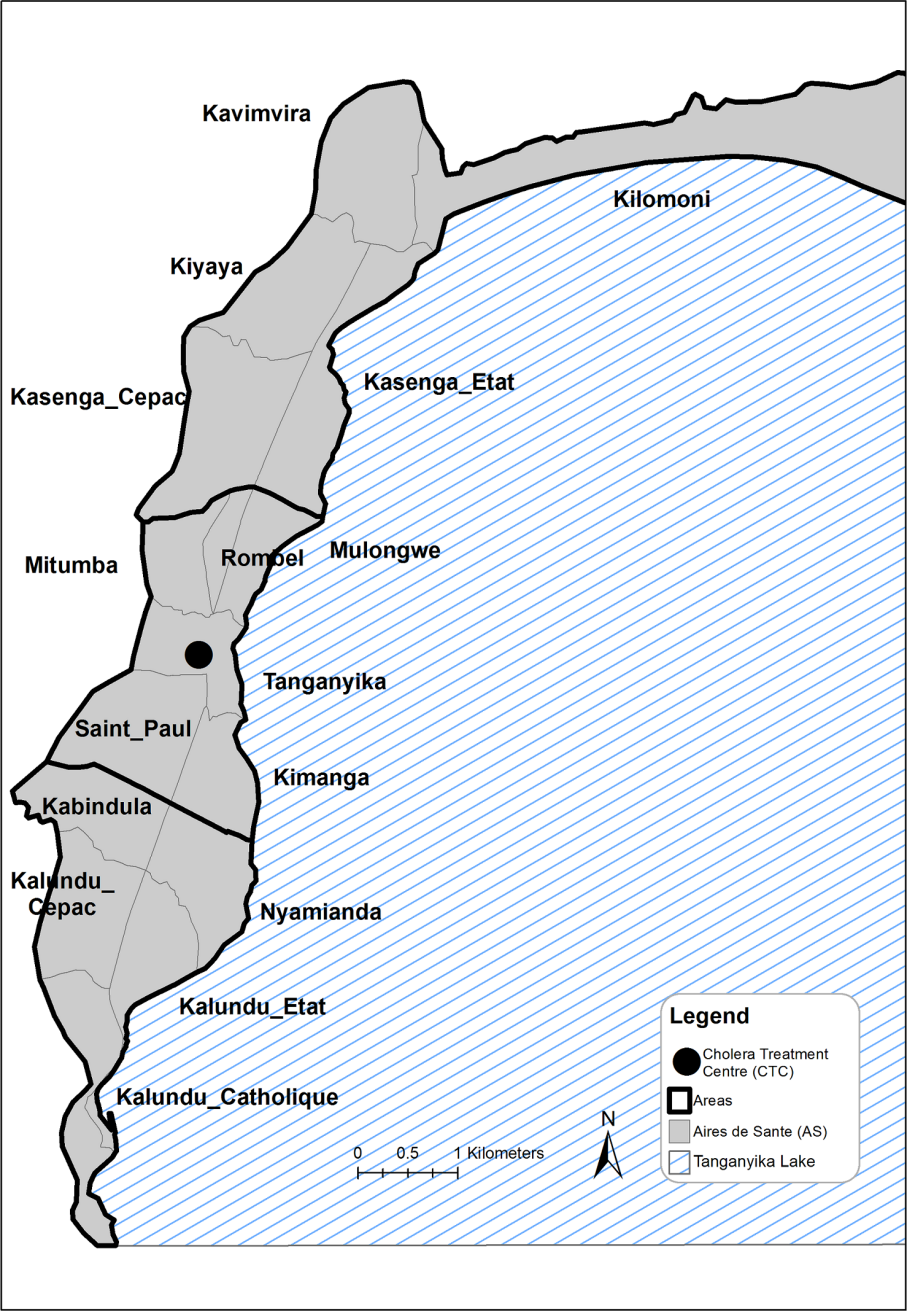
Map of Uvira.

The city of Uvira is located in South Kivu Province in eastern DRC on the shores of Lake Tanganyika and had an estimated population of 233,000 inhabitants in June 2016 (based on municipal authority figures). Uvira is divided into 16 neighbourhoods (“aires de santé” or AS), with estimated populations ranging from 6,500 to 24,000 people. These neighbourhoods can be grouped into three areas: North, Centre and South (Fig 1). The CTC at Uvira District Hospital admits all patients with acute diarrhoea, defined as 3 or more loose or liquid stools in 24h, presenting directly to the hospital or referred by local health posts. Patients are treated for dehydration and occasionally administered broad-spectrum antibiotics, zinc or albendazole. Admission and treatment at the CTC is free of charge for all patients. Patients admitted to the CTC may be referred to other hospital departments if they also present complicating medical conditions requiring additional care, such as diabetes, malaria or pregnancy.

At least one admission has been reported by the Uvira CTC to the District Health Office for every week except two between 2004 and 2017. All CTC admissions are considered as suspected cholera cases in Uvira and reported as such to the national level.

### Data and sample collection and laboratory analysis

From April 2016, a laboratory technician at Uvira District Hospital was trained to collect a rectal swab from all consenting newly admitted patients present at the CTC during daily morning visits 7 days per week. Demographic and clinical information was collected from the patient or a family member and the CTC patient register. The number of hours between first administration of antibiotic treatment (single-dose 300mg doxycycline) and sampling was also recorded.

The rectal swab was enriched in 4 ml of Alkaline Peptone Water (APW) for approximately six hours at ambient temperature (approximately 25°C +/- 2°C), and the apical portion of the unshaken APW vial was then tested for cholera with a rapid diagnostic test (RDT) (Crystal VC, Span Diagnostics, Surat, India), according to the manufacturer’s instructions.

Although stool samples are recommended by the manufacturer, rectal swabs were preferred to stool samples to reduce the risk of sample contamination, either across patients or with disinfectant, and to limit stool handling by clinical staff. The use of Crystal VC RDTs on enriched rectal swabs was however evaluated to have a sensitivity and specificity of 92% and 91% respectively in a previous study [11]. The enrichment step in APW has been shown to improve significantly the specificity of VC Crystal RDTs estimated at 71% when used directly on stool samples [12].

### Data collection

Data was collected electronically using Open Data Kit (ODK) on an Android tablet and stored remotely on a dedicated ODK server at the London School of Hygiene and Tropical Medicine [13]. Complementary information post-confirmation, such as discharge date and outcome were added later from the CTC patient register. Population estimates for each AS were obtained from Uvira Town Hall in June 2016 and, in the absence of estimates at other timepoints, populations were assumed to be stable over the study period. Average daily rainfall over the Uvira area was estimated using data from the Goddard Earth Sciences Data and Information Services Center (GES DISC) [14].Weekly rainfall was then calculated and each week categorized as dry or rainy with the median weekly rainfall of 3.6mm used as the cut-off. Weeks classified as “rainy” coincided mostly with the definition of rainy season for the area, between the months of October and May with an interruption in January [15].

### Data analysis

Data was analysed with STATA 14 (StataCorp, College Station, TX) and R [16].

### Case definitions

In this study, all patients admitted to the Uvira CTC were considered as suspected cholera cases, as per the definition recommended by the Global Task Force for Cholera Control (GTFCC) once an outbreak is declared [17]. They are referred to as suspected cases or admitted patients interchangeably. The same set of recommendations for cholera surveillance defines confirmed cholera cases as any suspected case from which Vibrio cholerae O1 or O139 is isolated. As no isolation of cholera strains were performed during the present study, we refer to participants tested positive with cholera RDTs as RDT-positive cases.

### Characteristics of RDT-positive cholera cases

Univariable logistic regression was used to assess whether age, sex, antibiotics administration before sampling, weekly number of admissions, area of residence, weekly rainfall category and clinical outcome (duration of stay and outcome of admission) were associated with the probability of a patient being enrolled or RDT positive. Predictors that were associated (p<0.1) in univariable models were included in a multivariable model and 2-way interactions were assessed by comparing likelihood ratios of models with or without interaction term. The final multivariable model was used to predict the proportion of untested patients that would have been confirmed as cholera cases by RDT. In order to investigate geographical variation in cholera confirmation rates at a smaller scale, AS of residence was introduced in the final model as a random effect to avoid coefficient estimate bias attributable to a high number of parameters [18]. Associations were reported as crude odds ratios (OR) for the univariable models, or adjusted odds ratios (aOR) for the multivariable models, along with 95% confidence intervals (CI).

### Time and space distribution of suspected and RDT-positive cholera cases

Weekly admissions per area and per AS were calculated over the entire study period and plotted. The number and proportion of weeks with suspected and RDT-positive cases was used as an indicator of the endemicity of suspected and RDT-positive cholera. Annual numbers of CTC admissions per area or AS were estimated as the mean number of admissions over 52 consecutive weeks (32 overlapping periods covered by the study), and annual number of RDT-positive cholera cases were derived by applying the confirmed/admission ratios estimated from the multivariable model for each area or AS.

### Funding and ethical considerations

This study was funded by the Agence Française de Développement and the Veolia Foundation. The funders had no role in study design, data collection and analysis, decision to publish, or preparation of the manuscript.

Demographic and clinical data for each patient is routinely collected in the CTC patient registry. Patients were only enrolled for RDT confirmation once a written informed consent was obtained, from either the patient or from the legal guardian for patients aged under 15. If illiterate, an oral consent was obtained and witnessed by an impartial third party. All data was anonymized before collection and analysis. In order to ensure that clinical staff deliver the same standard of care for all patients, the results of individual RDT results were not communicated to the clinical team.

The study was approved by the ethics committees of the School of Public Health at the University of Kinshasa, Democratic Republic of the Congo (ESP/CE/088c/2017), and of the London School of Hygiene and Tropical Medicine, United Kingdom (No 10603).

## Results

### Characteristics of admitted patients

**Fig 2:**
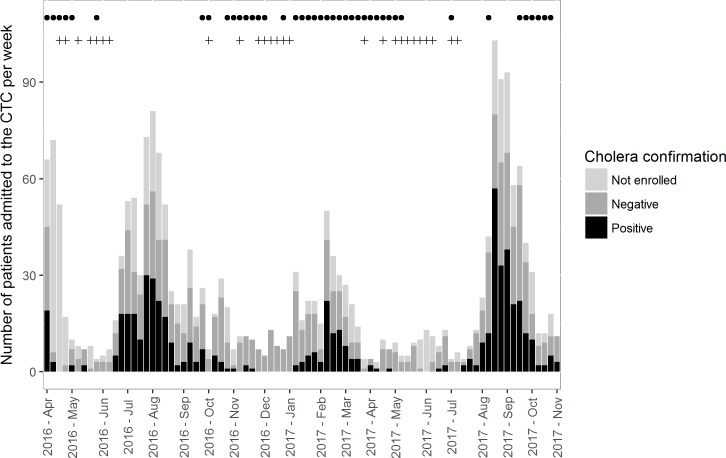
Weekly number of patients admitted to the CTC during the study period. The dots represent weeks classified as “rainy” (weekly rainfall estimated to be above 3.6 mm). The plus signs represent weeks with no cholera confirmed cases.

Between the 4^th^ of April 2016 and the 5^th^ of November 2017 (83 weeks), a total of 2,059 patients were admitted to the CTC. A majority (54.6%) of patients admitted to the CTC were 16 or older, while 336 (16.3%) were under 5 years of age. Similar numbers of female and male patients were admitted (1,019 and 1,038 respectively). Weekly admissions to the CTC ranged from 3 to 103 patients per week, with a median of 15 admissions per week (Fig 2). The 83 weeks of the study period were stratified into 4 categories of admission incidence–low, moderate, high and very high—defined by arbitrary cut-offs at 10, 20 and 50 admissions per week. These categories represented respectively 156 (7.6%), 302 (14.7%), 571 (27.7%) and 1,030 (50%) admissions over respectively 22 (26.5%), 22 (26.5%), 19 (22.9%) and 15 (18.1%) weeks. Date of exit and clinical outcome were missing for 9.3% and 7% of the admitted patients respectively. A majority (52.7%) of the patients admitted to the CTC stayed one or two nights, while 17.2% did not stay overnight and 30.1% stayed for 3 nights or more. The vast majority (87.1%) of the patients were discharged from the CTC, but 5.4% of the patients were transferred to other hospital departments. 10 deaths were recorded at the CTC during the study period, representing a case fatality ratio (CFR) of 0.5%.

### Enrolment and characteristics of enrolled patients

Rectal swabs were collected from 1,419 patients (68.9% of all those admitted). Of the 640 patients not enrolled, 39.5% refused to participate and 60.5% were not recruited due to their short stay at the CTC or the absence of the laboratory technician. The distribution of age and sex was similar among those recruited and not recruited. Patients admitted during weeks of very high admission incidence were slightly less likely to be enrolled in the study than patients admitted during other weeks. Similarly, patients leaving on the same day as admission were less likely to be enrolled, as were transferred or deceased patients. Characteristics of admitted and enrolled patients are summarized in Table 1.

**Table 1:** Characteristics of patients admitted to the CTC and enrolled into the confirmation study.

	Admitted	Enrolled
	n (% of total)	n (% enrolled)
**Total**	2059 (100%)	1419 (68.9%)
**Sex**		
Male	1038 (50.4%)	713 (68.7%)
Female	1019 (49.5%)	705 (69.2%)
Missing	2 (0.1%)	1 (50%)
**Age group**		
Under 2 year old	62 (3%)	37 (59.7%)
2 to 4 year old	274 (13.3%)	182 (66.4%)
5 to 15 year old	594 (28.8%)	412 (69.4%)
16 and older	1124 (54.6%)	787 (70%)
Missing	5 (0.2%)	1 (20%)
**Weekly admissions incidence**		
Low (3 to 9 admissions)	156 (7.6%)	109 (69.9%)
Moderate (10 to 19 admissions)	302 (14.7%)	208 (68.9%)
High (20 to 49 admissions)	571 (27.7%)	428 (75%)
Very High (50 to 103 admissions)	1030 (50%)	674 (65.4%)
**Duration of stay**		
Less than 24h	160 (7.8%)	84 (52.5%)
1 to 2 nights	1086 (52.7%)	771 (71%)
3 to 4 nights	501 (24.3%)	357 (71.3%)
5 nights or more	120 (5.8%)	81 (67.5%)
Missing	192 (9.3%)	126 (65.6%)
**Outcome**		
Discharged	1793 (87.1%)	1267 (70.7%)
Transferred	111 (5.4%)	47 (42.3%)
Death	10 (0.5%)	3 (30%)
Missing	145 (7%)	102 (70.3%)
**Area of residence**		
North (est. population 75,046)	891 (43.3%)	649 (72.8%)
Centre (est. population 98,384)	474 (23%)	316 (66.7%)
South (est. population 59,733)	611 (29.7%)	395 (64.6%)
Outside Uvira or missing	83 (4%)	59 (71.1%)
**Weekly rain category**		
Dry (< = 3.6 mm)	1178 (57.2%)	857 (72.8%)
Rainy (>3.6mm)	881 (42.8%)	562 (63.8%)

### Cholera confirmation

562 out of 1,419 rectal swabs (39.6%) were positive for cholera O1 after enrichment. None of the patients enrolled reported having taken antibiotics between symptom onset and CTC admission, and only 30 patients had been administered a single dose of doxycycline within the 12 hours preceding rectal sampling. Although *Vibrio cholerae* shedding was unlikely to have been affected in such a short time frame, antibiotics administration before sampling was included as a potential predictor in the analysis [19]. The proportions of positive samples by age, sex, weekly incidence category, antibiotics administration, clinical outcome, area of residence and weekly rainfall category are shown in Table 2, along with crude and adjusted odds ratios from the univariable and multivariable models.

**Table 2:** Predictors for cholera confirmation amongst patients admitted to the CTC.

	Enrolled (N = 1'419)	Positive	Univariable logistic regression		Multivariable logistic regression	
	n	n (% positive)	Crude OR (95% CI)	p-value*	Adjusted OR (95% CI)	p-value*
**Total**	1419	562 (39.6%)				
**Sex**						
Male	713	289 (40.5%)	1.08 (0.87–1.33)	*0*.*49*		
Female	705	273 (38.7%)	reference			
Missing	1	0 (0%)				
**Age group**						
Under 2 year-old	37	7 (18.9%)	0.38 (0.17–0.88)	*0*.*007*		
2 to 4 year old	182	81 (44.5%)	1.32 (0.95–1.82)			
5 to 15 year old	412	176 (42.7%)	1.23 (0.96–1.57)			
16 and older	787	298 (37.9%)	reference			
Missing	1	0 (0%)				
**Demographic group**						
Girls—under 2	16	6 (37.5%)	1.22 (0.44–3.44)	*p<0*.*001*	1.45 (0.49–4.3)	*p<0*.*001*
Girls—2 to 4 years	87	35 (40.2%)	1.37 (0.85–2.21)		1.21 (0.68–2.14)	
Girls—5 to 15 years	197	99 (50.3%)	2.08 (1.47–2.95)		2.12 (1.42–3.15)	
Women—16 and older	404	133 (32.9%)	reference		reference	
Boys—under 2	21	1 (4.8%)	0.1 (0.01–0.77)		0.12 (0.01–0.93)	
Boys—2 to 4 years	95	46 (48.4%)	1.91 (1.22–3.01)		1.85 (1.11–3.08)	
Boys—5 to 15 years	214	77 (36%)	1.15 (0.81–1.62)		1.04 (0.7–1.56)	
Men—15 and older	383	165 (43.1%)	1.54 (1.15–2.06)		1.78 (1.28–2.49)	
Missing	1	0 (0%)	excluded			
**Antibiotics treatment before sampling**						
No	1389	20 (1.4%)	0.76 (0.35–1.63)	*0*.*47*		
Yes	30	10 (33.3%)	reference			
**Weekly admissions incidence**						
Low (3 to 9 admissions)	109	14 (12.8%)	reference	*p<0*.*001*	reference	*p<0*.*001*
Moderate (10 to 19 admissions)	208	44 (21.2%)	1.82 (0.95–3.5)		1.83 (0.87–3.83)	
High (20 to 49 admissions)	428	155 (36.2%)	3.85 (2.13–6.98)		3.35 (1.69–6.66)	
Very High (50 to 103 admissions)	674	349 (51.8%)	7.29 (4.08–13.03)		5.33 (2.71–10.5)	
**Duration of stay**						
Less than 24h	84	26 (31%)	reference	*p<0*.*001*	reference	*p<0*.*001*
1 to 2 nights	771	227 (29.4%)	0.93 (0.57–1.52)		0.87 (0.5–1.53)	
3 to 4 nights	357	204 (57.1%)	2.97 (1.79–4.94)		2.44 (1.36–4.4)	
5 nights or more	81	53 (65.4%)	4.22 (2.2–8.1)		3.5 (1.67–7.34)	
Missing	126	52 (41.3%)	excluded			
**Outcome**						
Discharged	1267	493 (38.9%)	reference	*0*.*05*	reference	*0*.*02*
Transferred	47	25 (53.2%)	1.78 (0.99–3.2)		2.33 (1.14–4.77)	
Death	3	3 (100%)	excluded		excluded	
Missing	102	41 (40.2%)				
**Area of residence**						
North (est. population 75,046)	649	275 (42.4%)	1.5 (1.13–1.99)	*0*.*035*	1.57 (1.14–2.17)	*0*.*05*
Centre (est. population 98,384)	316	104 (32.9%)	reference		reference	
South (est. population 59,733)	395	157 (39.7%)	1.35 (0.99–1.84)		1.3 (0.91–1.86)	
Outside Uvira or missing	59	26 (44.1%)	1.61 (0.91–2.83)		1.57 (0.82–3.01)	
**Weekly rain category**						
Dry (< = 3.6 mm)	857	375 (43.8%)	1.56 (1.25–1.95)	*p<0*.*001*	1.34 (1.01–1.78)	*0*.*04*
Rainy (> 3.6 mm)	562	187 (33.3%)	reference		reference	

* χ2 significance test of likelihood ratio (LR test)

Although sex did not appear to be associated independently with cholera confirmation (p = 0.49), there was evidence for interaction between sex and age group (p<0.001). Girls aged 5 to 15 (OR 2.1; 95% CI: 1.5–3), boys aged 2 to 4 (OR 1.9; 95% CI: 1.2–3) and men aged 16 or older (OR 1.5; 95% CI: 1.1–2) were more likely to test positive than women aged 16 or older. Boys under 2 years of age were much less likely to test positive than women aged 16 or older (OR 0.1; 95% CI: 0.01–0.8). The proportion of positive samples was higher in weeks with higher numbers of admissions to the CTC, with the odds of a patient testing positive being more than 7-fold higher in very high incidence weeks compared to low incidence weeks. Similarly, the proportion of positive samples increased with the patient’s duration of stay. Patients staying 5 nights or more had more than 4 times the odds of testing positive compared to those leaving on the day of admission. All three patients tested who later died were confirmed cholera cases. There was weak evidence in univariable logistic regression analyses that transferred patients were more likely to be positive than discharged patients. Finally, patients admitted during dry weeks had higher odds of testing positive to cholera than those admitted during rainy weeks.

In a multivariable model including demographic group (combination of age and sex), admissions incidence category, duration of stay and outcome, area of residence and weekly rainfall category, girls aged 5 to 15, boys between 2 and 4 years of age and men aged 16 or older had respectively 2.1 (95% CI 1.4–3.2), 1.9 (95% CI 1.1–3.1) and 1.8 (95% CI 1.3–2.5) higher odds of testing positive compared to women aged 16 or older. Boys under 2 had 10 times lower odds of testing positive (aOR 0.1; 95% CI: 0.01–0.9). Patients admitted during weeks of high and very high admissions incidence had 3.4 (95% CI 1.7–6.7) and 5.3 (95% CI 2.7–10.5) times the odds respectively of testing positive compared to those admitted during low admissions weeks. Similarly, patients staying 3 or 4 nights or more than 5 nights had 2.4 (95% CI 1.4–4.4) and 3.5 (95% CI 1.7–7.3) times higher odds of being confirmed respectively than patients leaving on the day of admission. Patients transferred to another department were also more likely to be confirmed than discharged patients (aOR = 2.3; 95% CI 1.1–4.7). Patients residing in the North area of town were also more likely to be confirmed as cases than patients living in other areas of town (aOR = 1.6; 95%CI 1.1–2.2 compared to patients living in the centre), while patients admitted during dry weeks were more likely to be confirmed than during other weeks (aOR = 1.4; 95% CI 1–1.8).

There was no evidence of any 2-way interactions between demographic group, admissions category, clinical outcomes, area of residence and weekly rainfall. There was no evidence of a variation in confirmation rates amongst CTC admitted patients across AS after accounting for area (Likelihood ratio test of multivariable logistic model with AS as random effect vs multivariable model p = 1).

We used the multivariable model to predict the probability that non-enrolled patients would have tested positive. An estimated 39.6% of the 563 non-enrolled patients with complete information (age and sex group, duration of stay and outcome) are predicted to have been “true” cholera cases, very similar to the proportion of positives among those enrolled. The proportion of “true” cholera cases amongst all admissions (excluding 77 with incomplete information) is thus estimated to be 39.5% over the study period.

#### Time and space distribution of CTC admissions and RDT-positive cholera cases

1,976 patients admitted to the CTC over the study period reported residing in one of the 16 neighbourhoods of Uvira. Information on neighbourhood of residence was missing for 3 patients and 80 resided outside of Uvira municipality. For the entire city, this represented an annual incidence rate of 41.8 and 16.3 suspected and confirmed cases per 10,000 respectively (Table 3). Over the study period, weekly admissions incidence ranged from 0.13 to 4.3 per 10,000, with a median of 0.64 per 10,000.

**Table 3:** CTC admissions and RDT-positive cholera incidence rates and endemicity by neighbourhood and area of residence.

	Estimated population in June 2016	Number of admissions / patients enrolled / RDT-positive over study period	Confirmation ratio estimated from multivariable model	Annual CTC admissions / confirmed cholera incidence* per 10'000	Distribution of weekly CTC admissions incidence per 10'000 over study period		Number of weeks with at least one admission	Number of weeks with patients enrolled / at least one confirmed case
					Median (IQR)	Maximum	n (% over study period)	
AS Kasenga_Cepac	19182	241 / 158 / 60	41.6%	63.5 / 26.4	0.52 (0.52–2.09)	7.82	64 (77%)	46 / 25
AS Kasenga_Etat	24200	210 / 159 / 71	44.9%	46 / 20.6	0.41 (0.21–1.24)	6.2	62 (75%)	54 / 30
AS Kavimvira	13959	195 / 156 / 78	45.1%	70.6 / 31.8	0.72 (0–1.43)	24.36	51 (61%)	43 / 23
AS Kilomoni	11162	164 / 117 / 38	38.4%	83.7 / 32.1	0.9 (0–1.79)	12.54	53 (64%)	40 / 20
AS Kiyaya	6543	81 / 59 / 28	41.6%	65.4 / 27.2	0 (0–1.53)	25.98	34 (41%)	27 / 16
**NORTH**	**75046**	**891 / 649 / 275**	**42.6%**	**62.3 / 26.6**	**0.8 (0.4–1.6)**	**10.66**	**81 (98%)**	**73 / 50**
AS Mitumba	19610	137 / 24 / 5	30.9%	39.5 / 12.2	0.51 (0–1.02)	6.63	47 (57%)	42 / 19
AS Mulongwe	12815	98 / 90 / 32	32.7%	40.8 / 13.4	0 (0–1.56)	5.46	38 (46%)	32 / 14
AS Kimanga	13879	35 / 72 / 19	30.6%	12.9 / 3.9	0 (0–0.72)	2.88	22 (27%)	18 / 4
AS RombeI	15092	53 / 35 / 14	32.6%	15.3 / 5	0 (0–0.66)	3.98	26 (31%)	21 / 12
AS Saint_Paul	21114	95 / 61 / 22	31.9%	25.6 / 8.2	0.47 (0–0.95)	3.32	51 (61%)	36 / 13
AS Tanganyika	15874	56 / 34 / 12	29.7%	20.6 / 6.1	0 (0–0.63)	2.52	36 (43%)	26 / 11
**CENTRE**	**98384**	**474 / 316 / 104**	**31.5%**	**26.2 / 8.2**	**0.41 (0.2–0.81)**	**2.54**	**74 (89%)**	**68 / 37**
AS Kabindula	15525	149 / 83 / 33	42.4%	42.8 / 18.1	0 (0–1.29)	12.88	39 (47%)	35 / 14
AS Kalundu_Catholique	7845	50 / 41 / 14	34.5%	36.4 / 12.5	0 (0–1.27)	5.1	36 (43%)	31 / 12
AS Kalundu_Cepac	10783	180 / 103 / 42	41.4%	82.9 / 34.3	0.93 (0–1.85)	22.26	51 (61%)	40 / 20
AS Kalundu_Etat	14378	160 / 121 / 54	39.4%	60.8 / 24	0.7 (0–2.09)	7.65	53 (64%)	43 / 24
AS Nyamianda	11202	72 / 47 / 14	38.9%	36.8 / 14.3	0 (0–0.89)	4.46	35 (42%)	29 / 11
**SOUTH**	**59733**	**611 / 395 / 157**	**40.3%**	**52.4 / 21.1**	**0.67 (0.33–1.34)**	**9.38**	**78 (94%)**	**69 / 37**
Total Uvira municipality	248476	1976 / 1360 / 536	39.4%	41.8 / 16.5	0.64 (0.34–1.27)	4.29	83 (100%)	81 / 57
Unknown residence location or outside Uvira		83 / 59 / 26						

* based on the mean number of admissions over 52 consecutive weeks (32 periods) and the confirmed/admission ratios estimated by the multivariable logistic regression mode

The northern area had the highest annual incidence of both suspected and confirmed cases, 62.3 and 26.6 cases per 10,000 respectively. At the AS level, the highest annual incidence rate for suspected and confirmed cases were found in AS Kalundu Cepac (South), AS Kavimvira and AS Kilomoni (North). In a given week, the highest weekly incidence rate for admissions by AS over the entire study period was found in AS Kiyaya (North), with 26 cases per 10,000 per week, followed closely by AS Kavimvira and AS Kalundu Cepac. The lowest annual incidence rates by AS for both suspected and confirmed cases were found in AS Kimanga and AS Rombe I (Centre).

At least one admission to the CTC was recorded every week throughout the study, with at least one RDT-positive case in more than two thirds of the weeks. At the AS level, the proportion of weeks with admissions ranged from 27% for AS Kimanga, to 77% for AS Kasenga Cepac.

**Fig 3:**
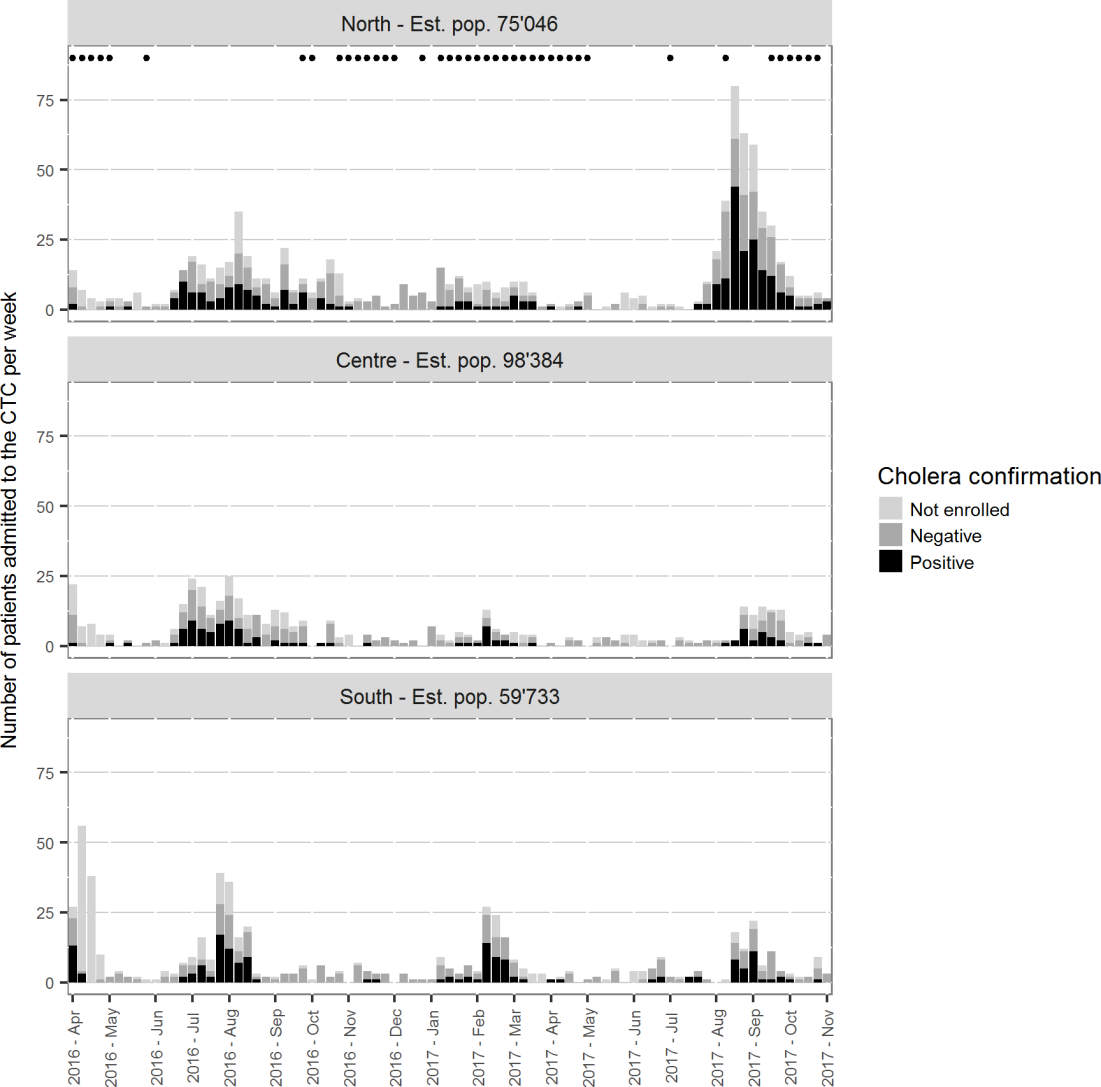
Weekly number of suspected, RDT-positive and RDT-negative cholera cases admitted to the CTC, by area of residence.

The distribution of cases over time shows similar patterns over the three areas of Uvira, although with different amplitudes in variations in the number of both suspected and RDT-positive cases (Fig 3). The number of AS contributing admissions in any given week ranged from 3 to 16 (median 8 AS affected, IQR 5–11 AS). Patients resided in 12 to 16 AS during the weeks in the very high admissions category.

## Discussion

The epidemiology of cholera, particularly in endemic areas, is often described based on suspected rather than confirmed cases despite the lack of specificity of the standard case definition. This is likely to limit our understanding of cholera transmission and of how cholera prevention and outbreak response activities should be designed.

Using rapid diagnostic tests to confirm cholera amongst patients admitted to the Uvira CTC over an eighteen-month period, this study reveals that only about 40% of patients admitted to the CTC were confirmed as infected with cholera, giving an estimated city-wide incidence of confirmed cholera of sufficient severity to seek healthcare at the CTC of 16.5 cases per 10,000 annually. The data show an increasing probability of confirmed cholera when CTC admission rates are higher, with the proportion of confirmed cases reaching 52% during weeks with 50 CTC admissions or more. The data also suggest the occurrence of two distinct peaks each year, one around weeks 10 to 15 (March–April), and one around weeks 30 to 38 (August to October) of the calendar year. During these peaks, incidence of both admissions and suspected cases increased across all three areas of Uvira, although to a different degree. Both the incidence of CTC admissions and the case confirmation ratio were higher during weeks with lower rainfall. This study however confirms the endemic nature of cholera in Uvira, with confirmed cholera cases identified during 70% of the weeks observed—57 out of 81 with at least one patient enrolled. The incidence of CTC admissions, case confirmation ratio and the number of weeks with suspected and confirmed cases are all higher in the northern area of town, compared to the central area, and to a lesser degree to the southern area. This study also shows that at the individual level, female patients aged 5 to 15, male patients between 2 and 4 and over 15 years of age, patients admitted for 3 nights or more, and patients transferred to other departments are more likely to be RDT-positive. Boys under two years of age are however less likely to test positive with RDT.

The incidence rates of suspected and RDT-positive cholera cases as well as the confirmation ratio observed in this study are broadly similar to those reported for the Goma enhanced surveillance site of the AFRICHOL study, where annual incidence rates for suspected / confirmed cholera were estimated to 40.5 / 13.6 per 10,000 and the confirmation ratio to 50% [4]. Although estimated over a larger geographical area, suspected cholera incidence rates in the most affected province of Mozambique (Cabo Delgado) in 2009–2010 were much lower than those in Uvira with 14.2 cases per 10,000 per year, while cumulative suspected cholera incidence rates over 6 years (2011–2016) in cholera “hot spot” districts in Uganda did not exceed 100 per 10,000, suggesting an average annual rate of 16.7 suspected cases per 10,000 [20, 21]. The confirmed cholera incidence rate in Kolkata, India, was broadly similar to that observed here, with 22 confirmed cases per 10,000 per year[22]. Suspected cholera incidence rates in Uvira are however of a much lower magnitude than those observed during the first year of the Haiti cholera epidemic that started in October 2010, where the yearly incidence rate of suspected cholera reached 3,000 per 10,000 in some districts, and 490 per 10,000 over the entire country [23].

The higher incidence of both suspected and confirmed cases during the dry season observed in our study is in contrast to findings by Bompangue et al. in the region [9]. Data collected between 2002 and 2008 in Uvira showed a positive correlation between rainfall and number of suspected cholera cases reported in Uvira with two to five weeks latency. However, these results are not incompatible with a previous study showing that tap water supply interruptions were associated with a 2.5-fold increase in admissions to the CTC in the 12 following days as these tap water supply interruptions are longer and more frequent during the dry season due to more power outage [24]. Further investigations of seasonal patterns of admissions and RDT-positive cases, in relation with tap water availability and tap water supply interruptions will require specific spatio-temporal methods and a longer dataset.

Our study has various limitations. First, it is based on passive surveillance data collected at a healthcare facility. It is therefore limited to those seeking health care, and this decision is likely to depend on several factors including the perceived severity of symptoms, the monetary and opportunity costs of seeking care, and the perceived quality of care provided at the CTC. Even though all health posts and community health workers across town are instructed to refer acute diarrhoea cases to the CTC, where patients are treated for free, it is not possible to exclude the possibility that some groups of the population choose not to seek treatment if symptoms are deemed non-severe, or to seek treatment elsewhere in a private facility or from traditional healers. Healthcare seeking behaviours may also vary over time, especially during an outbreak. Further, it is likely that CTC admission criteria may be more stringent during an outbreak when capacity is already stretched. It is also worth noting that the clinical presentation of cholera infection can vary from no symptoms at all, to mild and short-lasting gastro-intestinal symptoms and, in less than 10% of the cases, to severe diarrhoea with vomiting leading rapidly to dehydration and sometimes death [25]. Although it is accepted that symptom severity–and therefore the probability of a case being admitted to a health facility—is dependent on the inoculum ingested, infectious dose is influenced by a number of factors such as hypochlorhydria, concurrent ingestion with food, retinol A deficiency or blood group O [26–28]. Thus, clinical cholera illness incidence rates are only a proxy for the level of exposure of an individual to *Vibrio cholerae* and intensity of cholera transmission within a community.

Another important limitation of this study is the way cholera cases were confirmed. Although the use of RDTs on stool samples after an enrichment stage has been shown to perform as well as traditional culture methods, the sensitivity and specificity of this method were 86% and 100% respectively in comparison with PCR in another study [29]. The amount of stool collected with a rectal swab may also be less standardized, and the use of enriched rectal swabs with Crystal VC RDTs had a sensitivity and specificity of 92% and 91% respectively in comparison with culture methods [11]. Although WHO’s standard definition of a confirmed cholera case is based on the isolation of *Vibrio cholerae* O1 or O139 in a suspected case’s stools, this is not sufficient to attribute diarrhoea aetiology to cholera only, as acute diarrhoea may actually be caused by other enteric infections concurrently present, or by non-infectious causes. Asymptomatic cholera infections and associated *Vibrio cholerae* shedding in stools are common in endemic areas [26, 30]. A case-control or longitudinal study design would be needed to estimate in a robust manner the true cholera attributable diarrhoea burden in Uvira CTC patients. Finally, these results stem from only 69% of the patients admitted, and although those enrolled do not appear to differ importantly from those not participating on measured socio-demographic and clinical factors, selection bias associated with unmeasured factors amongst those enrolled cannot be excluded.

Our study confirms that cholera is indeed a public health concern in Uvira. Our findings substantiate the endemicity and incidence of cholera, with RDT-positive cholera cases admitted to the CTC nearly all year round, and from all neighbourhoods. Although a non-specific case definition is valuable for timely and sensitive outbreak detection, they highlight that a substantial proportion of suspected cases are not testing positive with RDTs, and that confirmation of suspected cases is needed to understand the true burden of cholera and to investigate risk factors for cholera, especially in endemic areas. This need to improve cholera surveillance with a more specific case definition was recently highlighted by another study in seven African countries [5]. This would also contribute to the planning, targeting and evaluation of cholera vaccine campaigns. In Uvira, deployment of an oral cholera vaccine alone would only address a fraction of the diarrhoea cases admitted to the CTC which are currently suspected to be cholera cases. Our study also demonstrates the feasibility of enhanced surveillance of cholera with RDTs over 18 months under challenging circumstances and limited human resources. Although a different clinical management is not called for, the high proportion of negative results amongst CTC admitted patients underlines as well the importance of high quality infection prevention and control (IPC) measures, to avoid cholera-negative patients becoming infected with cholera during their stay at the CTC. This is also true for patients transferred to other hospital departments–often paediatrics or obstetrics—where there may be less stringent infection control measures in place. The high proportion of negative results also raises the question of non-cholera aetiology of diarrhoea in CTC admitted patients, and of the burden attributable to other entero-pathogens that could potentially require specific control measures, such as rotavirus vaccination. Further, we found that only boys younger than 2 are less likely to be confirmed as cholera cases than patients from other older age groups, which raises questions about the exclusion of under 2s from the WHO suspected case definition outside of an outbreak [3].

The tap water supply improvements currently being implemented in Uvira provide an opportunity to study the effect of such improvements on both cholera and non-cholera diarrhoea. Exploration of time and space clustering of suspected and confirmed cases, in relation to the changes in tap water access and reliability, may provide explanations for the substantial variations in incidence rates observed between different areas of town and over time. An impact evaluation of these tap water supply improvements in Uvira is under way [31]. In addition, identification of other entero-pathogens shed by suspected cases will provide a better understanding of the non-cholera diarrhoeal aetiology in patients admitted to the CTC. The planned genomic characterization of cholera strains isolated from confirmed cholera cases in Uvira will provide insight into the regional dynamics of cholera transmission.

In summary, our data show that cholera is endemic throughout Uvira, but only 40% of admissions to Uvira CTC have a detectable cholera infection. Interventions aimed at reducing the burden of cholera and other diarrhoeal diseases should be a public health priority.

## Supporting information

S1 Table STROBE checklist. (DOC)

S2 Table Dataset. (CSV)
