# Supplementary material for: Confirmation of cholera by rapid diagnostic test amongst patients admitted to the cholera treatment centre in Uvira, Democratic Republic of the Congo
Source: PLoS One. 2018 Aug 1;13(8):e0201306. doi: 10.1371/journal.pone.0201306 (PMC6070262; doi:10.1371/journal.pone.0201306)
Supplement: S1 Table — (DOC) [file pone.0201306.s001.doc]

**STROBE Statement—Checklist of items that should be included in reports of *cross-sectional studies***

**Manuscript submitted to PloS NTD entitled “Laboratory confirmed cholera amongst patients admitted to the cholera treatment centre in Uvira, Democratic Republic of the Congo”**

**(PNTD-D-18-00167)**

|  | Item No | Recommendation | Manuscript Excerpt |
| --- | --- | --- | --- |
| **Title and abstract** | 1 | (*a*) Indicate the study’s design with a commonly used term in the title or the abstract | Title: “Laboratory confirmed cholera amongst patients admitted to the cholera treatment centre in Uvira, Democratic Republic of the Congo” |
|  |  | (*b*) Provide in the abstract an informative and balanced summary of what was done and what was found | Abstract: “Between April 2016 and November 2017, 69% of the 2,059 patients admitted to the Uvira Cholera Treatment Centre (CTC) were tested for cholera with rapid diagnostic tests. Of those admitted as suspected cholera cases, only 40% tested positive for cholera, equivalent to an estimated annual incidence of suspected/confirmed cholera in Uvira of 43.8 and 16.3 cases per 10,000 inhabitants respectively. A multivariable logistic regression indicates that adult men and girls aged 5 to 15 years are respectively 1.8 and 2.1 times more likely to test positive than adult women. The odds of testing positive also increase as weekly admissions to the CTC rise, with up to a 5-fold increase observed during the weeks with the highest numbers of admissions compared to the lowest ones. Other predictors of cholera confirmation include duration of stay at the CTC, clinical outcome of admission, lower weekly rainfall and area of residence in Uvira, with the northern part of town having the highest confirmation rate.” |
| Introduction | | |  |
| Background/rationale | 2 | Explain the scientific background and rationale for the investigation being reported | Introduction: “In 2016, more than 130,000 cases of cholera were reported to the World Health Organisation (WHO) globally. These figures are likely incomplete due to weak national surveillance systems and the actual global number of cholera cases is estimated to be closer to 3 million every year. Comprehensive surveillance is also hampered by the relatively non-specific clinical definition of suspected cholera cases, especially in endemic areas. WHO standard case definitions for suspected cases distinguish between areas where cholera is not known to be present and areas where there is an ongoing cholera outbreak, that is where at least one suspected case of cholera has been laboratory confirmed. In Haiti, the specificity of this case definition in an outbreak setting was estimated to be only 43% over a two-year study in 4 hospitals. Over-reporting of suspected cholera cases is therefore possible in areas with a long history of cholera outbreaks, when laboratory confirmation is only done on initial outbreak cases.” |
| Objectives | 3 | State specific objectives, including any prespecified hypotheses | End of introduction: “Our study describes the epidemiology of suspected and confirmed cholera based on admissions and laboratory confirmation data collected over 18 months at the Cholera Treatment Centre (CTC) of Uvira in eastern DRC.” |
| Methods | | |  |
| Study design | 4 | Present key elements of study design early in the paper | End of introduction: “Our study describes the epidemiology of suspected and confirmed cholera based on admissions and laboratory confirmation data collected over 18 months at the Cholera Treatment Centre (CTC) of Uvira in eastern DRC.” |
| Setting | 5 | Describe the setting, locations, and relevant dates, including periods of recruitment, exposure, follow-up, and data collection | All of the Methods section |
| Participants | 6 | (*a*) Give the eligibility criteria, and the sources and methods of selection of participants | Methods section: “From April 2016, a laboratory technician at Uvira District Hospital was trained to collect a rectal swab from all consenting newly admitted patients present at the CTC during daily morning visits 7 days per week.” |
| Variables | 7 | Clearly define all outcomes, exposures, predictors, potential confounders, and effect modifiers. Give diagnostic criteria, if applicable | Methods section, paragraphs “Characteristics of confirmed cholera cases” and “Time and space distribution of suspected and confirmed cholera cases” |
| Data sources/ measurement | 8* | For each variable of interest, give sources of data and details of methods of assessment (measurement). Describe comparability of assessment methods if there is more than one group | Methods section, paragraph “Data Collection”: “Data was collected electronically using Open Data Kit (ODK) on an Android tablet and stored remotely on a dedicated ODK server at the London School of Hygiene and Tropical Medicine. Complementary information post-confirmation, such as discharge date and outcome were added later from the CTC patient register. Population estimates for each AS were obtained from Uvira Town Hall in June 2016 and, in the absence of estimates at other timepoints, populations were assumed to be stable over the study period. Average daily rainfall over the Uvira area was estimated using data from the Goddard Earth Sciences Data and Information Services Center (GES DISC). Weekly rainfall was then calculated and each week categorized as dry or rainy with the median weekly rainfall of 3.6mm used as the cut-off. Weeks classified as “rainy” coincided mostly with the definition of rainy season for the area, between the months of October and May with an interruption in January.” |
| Bias | 9 | Describe any efforts to address potential sources of bias | Results section: “Rectal swabs were collected from 1,419 patients (68.9% of all those admitted). Of the 640 patients not enrolled, 39.5% refused to participate and 60.5% were not recruited due to their short stay at the CTC or the absence of the laboratory technician. The distribution of age and sex was similar among those recruited and not recruited. Patients admitted during weeks of very high admission incidence were slightly less likely to be enrolled in the study than patients admitted during other weeks. Similarly, patients leaving on the same day as admission were less likely to be enrolled, as were transferred or deceased patients. Characteristics of admitted and enrolled patients are summarized in Table 1.” |
| Study size | 10 | Explain how the study size was arrived at | Results section: “Between the 4th of April 2016 and the 5th of November 2017 (83 weeks), a total of 2,059 patients were admitted to the CTC.” And “Rectal swabs were collected from 1,419 patients (68.9% of all those admitted).” |
| Quantitative variables | 11 | Explain how quantitative variables were handled in the analyses. If applicable, describe which groupings were chosen and why | Methods section: “…Average daily rainfall over the Uvira area was estimated using data from the Goddard Earth Sciences Data and Information Services Center (GES DISC). Weekly rainfall was then calculated and each week categorized as dry or rainy with the median weekly rainfall of 3.6mm used as the cut-off. Weeks classified as “rainy” coincided mostly with the definition of rainy season for the area, between the months of October and May with an interruption in January.” And “Annual numbers of CTC admissions per area or AS were estimated as the mean number of admissions over 52 consecutive weeks (32 overlapping periods covered by the study), and annual number of confirmed cholera cases were derived by applying the confirmed/admission ratios estimated from the multivariable model for each area or AS.” |
| Statistical methods | 12 | (*a*) Describe all statistical methods, including those used to control for confounding | Methods section, paragraphs “Characteristics of confirmed cholera cases” and “Time and space distribution of suspected and confirmed cholera cases” |
|  |  | (*b*) Describe any methods used to examine subgroups and interactions | Methods section: “2-way interactions were assessed by comparing likelihood ratios of models with or without interaction term.” |
|  |  | (*c*) Explain how missing data were addressed | Methods section: “The final multivariable model was used to predict the proportion of untested patients that would have been confirmed as cholera cases” |
|  |  | (*d*) If applicable, describe analytical methods taking account of sampling strategy | NA |
|  |  | (*e*) Describe any sensitivity analyses | NA |
| Results | | |  |
| Participants | 13* | (a) Report numbers of individuals at each stage of study—eg numbers potentially eligible, examined for eligibility, confirmed eligible, included in the study, completing follow-up, and analysed | Results section: “Between the 4th of April 2016 and the 5th of November 2017 (83 weeks), a total of 2,059 patients were admitted to the CTC.” And “Rectal swabs were collected from 1,419 patients (68.9% of all those admitted).” |
|  |  | (b) Give reasons for non-participation at each stage | Results section: “Of the 640 patients not enrolled, 39.5% refused to participate and 60.5% were not recruited due to their short stay at the CTC or the absence of the laboratory technician.” |
|  |  | (c) Consider use of a flow diagram | NA |
| Descriptive data | 14* | (a) Give characteristics of study participants (eg demographic, clinical, social) and information on exposures and potential confounders | Available in tables 1 & 2 |
|  |  | (b) Indicate number of participants with missing data for each variable of interest | Available in tables 1 & 2 |
| Outcome data | 15* | Report numbers of outcome events or summary measures | Results section: “Rectal swabs were collected from 1,419 patients (68.9% of all those admitted).” And “562 out of 1,419 rectal swabs (39.6%) were positive for cholera O1 after enrichment.” |
| Main results | 16 | (*a*) Give unadjusted estimates and, if applicable, confounder-adjusted estimates and their precision (eg, 95% confidence interval). Make clear which confounders were adjusted for and why they were included | Available in Table 2 and in methods section “Predictors that were associated (p<0.1) in univariable models were included in a multivariable model” |
|  |  | (*b*) Report category boundaries when continuous variables were categorized | Available in Tables 1 & 2 |
|  |  | (*c*) If relevant, consider translating estimates of relative risk into absolute risk for a meaningful time period | NA |
| Other analyses | 17 | Report other analyses done—eg analyses of subgroups and interactions, and sensitivity analyses | NA |
| Discussion | | |  |
| Key results | 18 | Summarise key results with reference to study objectives | Discussion section: “Using rapid diagnostic tests to confirm cholera amongst patients admitted to the Uvira CTC over an eighteen-month period, this study reveals that only about 40% of patients admitted to the CTC were confirmed as infected with cholera, giving an estimated city-wide incidence of confirmed cholera of sufficient severity to seek healthcare at the CTC of 16.5 cases per 10,000 annually. The data show an increasing probability of confirmed cholera when CTC admission rates are higher, with the proportion of confirmed cases reaching 52% during weeks with 50 CTC admissions or more. The data also suggest the occurrence of two distinct peaks each year, one around weeks 10 to 15 (March – April), and one around weeks 30 to 38 (August to October) of the calendar year. During these peaks, incidence of both admissions and suspected cases increased across all three areas of Uvira, although to a different degree. Both the incidence of CTC admissions and the case confirmation ratio were higher during weeks with lower rainfall. This study however confirms the endemic nature of cholera in Uvira, with confirmed cholera cases identified during 70% of the weeks observed - 57 out of 81 with at least one patient enrolled. The incidence of CTC admissions, case confirmation ratio and the number of weeks with suspected and confirmed cases are all higher in the northern area of town, compared to the central area, and to a lesser degree to the southern area. This study also shows that at the individual level, female patients aged 5 to 15, male patients over 15 years of age, patients admitted for 3 nights or more, and patients transferred to other departments are more likely to be cholera confirmed.” |
| Limitations | 19 | Discuss limitations of the study, taking into account sources of potential bias or imprecision. Discuss both direction and magnitude of any potential bias | Discussion section: Our study has various limitations. First, it is based on passive surveillance data collected at a healthcare facility. It is therefore limited to those seeking health care, and this decision is likely to depend on several factors including the perceived severity of symptoms, the monetary and opportunity costs of seeking care, and the perceived quality of care provided at the CTC. Even though all health posts and community health workers across town are instructed to refer acute diarrhoea cases to the CTC, where patients are treated for free, it is not possible to exclude the possibility that some groups of the population choose not to seek treatment if symptoms are deemed non-severe, or to seek treatment elsewhere in a private facility or from traditional healers. Healthcare seeking behaviours may also vary over time, especially during an outbreak. Further, it is likely that CTC admission criteria may be more stringent during an outbreak when capacity is already stretched. It is also worth noting that the clinical presentation of cholera infection can vary from no symptoms at all, to mild and short-lasting gastro-intestinal symptoms and, in less than 10% of the cases, to severe diarrhoea with vomiting leading rapidly to dehydration and sometimes death [21]. Although it is accepted that symptom severity – and therefore the probability of a case being admitted to a health facility - is dependent on the inoculum ingested, infectious dose is influenced by a number of factors such as hypochlorhydria, concurrent ingestion with food, retinol A deficiency or blood group O [22-24]. Thus, clinical cholera illness incidence rates are only a proxy for the level of exposure of an individual to vibrio cholerae and intensity of cholera transmission within a community.  Another important limitation of this study is the way cholera cases were confirmed. Although the use of RDTs after an enrichment stage has been shown to perform as well as traditional culture methods, the sensitivity and specificity of this method were 86% and 100% respectively in comparison with PCR in another study [25]. Although WHO’s standard definition of a confirmed cholera case is based on the detection of Vibrio cholerae O1 in a suspected case’s stools, this is not sufficient to attribute diarrhoea aetiology to cholera only, as acute diarrhoea may actually be caused by other enteric infections concurrently present, or by non-infectious causes. Asymptomatic cholera infections and associated vibrio cholerae shedding in stools are common in endemic areas [22, 26]. A case-control or longitudinal study design would be needed to estimate in a robust manner the true cholera attributable diarrhoea burden in Uvira CTC patients. Finally, these results stem from only 69% of the patients admitted, and although those enrolled do not appear to differ importantly from those not participating on measured socio-demographic and clinical factors, selection bias associated with unmeasured factors amongst those enrolled cannot be excluded.” |
| Interpretation | 20 | Give a cautious overall interpretation of results considering objectives, limitations, multiplicity of analyses, results from similar studies, and other relevant evidence | All discussion section |
| Generalisability | 21 | Discuss the generalisability (external validity) of the study results | Discussion section: “Although a non-specific case definition is valuable for timely and sensitive outbreak detection, they highlight that a substantial proportion of suspected cases are not confirmed cases, and that laboratory confirmation of suspected cases is needed to understand the true burden of cholera and to investigate risk factors for cholera, especially in endemic areas.” |
| Other information | | |  |
| Funding | 22 | Give the source of funding and the role of the funders for the present study and, if applicable, for the original study on which the present article is based | Methods section: “This study was funded by the Agence Française de Développement and the Veolia Foundation. The funders had no role in study design, data collection and analysis, decision to publish, or preparation of the manuscript.” |

*Give information separately for exposed and unexposed groups.

**Note:** An Explanation and Elaboration article discusses each checklist item and gives methodological background and published examples of transparent reporting. The STROBE checklist is best used in conjunction with this article (freely available on the Web sites of PLoS Medicine at http://www.plosmedicine.org/, Annals of Internal Medicine at http://www.annals.org/, and Epidemiology at http://www.epidem.com/). Information on the STROBE Initiative is available at www.strobe-statement.org.
